# Role of microRNAs, circRNAs and long noncoding RNAs in acute myeloid leukemia

**DOI:** 10.1186/s13045-019-0734-5

**Published:** 2019-05-24

**Authors:** Yan Liu, Zhiheng Cheng, Yifan Pang, Longzhen Cui, Tingting Qian, Liang Quan, Hongyou Zhao, Jinlong Shi, Xiaoyan Ke, Lin Fu

**Affiliations:** 1 Department of Hematology, The Second Affiliated Hospital of Guangzhou Medical University, Guangzhou, 510260 China; 2 Translational Medicine Center, Huaihe Hospital of Henan University, Kaifeng, 475000 China; 3 Translational Medicine Center, The Second Affiliated Hospital of Guangzhou Medical University, Guangzhou, 510260 China; 4 Department of Pathology and Medical Biology, University Medical Center Groningen, University of Groningen, Groningen, Netherlands; 5 Department of Medicine, William Beaumont Hospital, Royal Oak, MI 48073 USA; 6 Department of Laser Medicine, Chinese PLA General Hospital, Beijing, 100853 China; 7 Department of Biomedical Engineering, Chinese PLA General Hospital, Beijing, 100853 China; 8 Department of Hematology and Lymphoma Research Center, Peking University Third Hospital, Beijing, 100191 China; 9 Department of Hematology, Huaihe Hospital of Henan University, Kaifeng, 475000 China

**Keywords:** Acute myeloid leukemia, microRNA, circRNA, Long noncoding RNA

## Abstract

Acute myeloid leukemia (AML) is a malignant tumor of the immature myeloid hematopoietic cells in the bone marrow (BM). It is a highly heterogeneous disease, with rising morbidity and mortality in older patients. Although researches over the past decades have improved our understanding of AML, its pathogenesis has not yet been fully elucidated. Long noncoding RNAs (lncRNAs), microRNAs (miRNAs), and circular RNAs (circRNAs) are three noncoding RNA (ncRNA) molecules that regulate DNA transcription and translation. With the development of RNA-Seq technology, more and more ncRNAs that are closely related to AML leukemogenesis have been discovered. Numerous studies have found that these ncRNAs play an important role in leukemia cell proliferation, differentiation, and apoptosis. Some may potentially be used as prognostic biomarkers. In this systematic review, we briefly described the characteristics and molecular functions of three groups of ncRNAs, including lncRNAs, miRNAs, and circRNAs, and discussed their relationships with AML in detail.

## Background

Acute myeloid leukemia (AML) is an aggressive hematological malignancy characterized by abnormal proliferation and differentiation of the immature myeloid cells [1]. Despite a growing list of treatment options, most patients still relapse and die after remission, and the prognosis remains unideal [2]. It is necessary to explore new biomarkers for diagnosis, prognostication, and therapeutic targets of AML so as to develop more effective surveillance and treatment programs.

The discovery of noncoding RNAs (ncRNAs) opens up new prospects for AML diagnosis, prognosis and treatment. ncRNAs are functional small RNA molecules that are not translated into a protein [3]. The DNA molecules that make up the human genome are about 3 billion base pairs, of which about 5–10% are stably transcribed, but protein-coding genes account for less than 2% of the human genome. The remaining 3–8% of the genome are transcribed into non-coding transcripts, i.e., ncRNAs [4–6]. ncRNAs are divided into two categories based on their functions: housekeeping and regulatory, the latter includes miRNAs, circRNAs, and lncRNAs. Regulatory ncRNAs are extensively involved in gene transcription and translation. They are key players in physiological and pathological processes such as cell differentiation, ontogenesis, inflammation, and angiogenesis. There is emerging evidence that miRNAs, circRNAs, and lncRNAs actively participate in the pathogenesis of major hematological malignancies including AML [7]. In this review, we aimed to provide a comprehensive summary of the roles of miRNAs, circRNAs, and lncRNAs in AML, and to illustrate their diagnostic and prognosticating potentials in this disease.

## MicroRNA

MicroRNAs (miRNAs) are small RNA molecules of approximately 22 nucleotides that bind to the 3′-untranslated region (3′-UTR) of the target mRNA and negatively regulate the expression of the target gene at the transcriptional level [8]. miRNAs mainly participate in the pathogenesis of AML through the following five mechanisms: copy number alterations, change in the proximity to the oncogenic genomic region due to chromosomal translocation, epigenetic changes, aberrant targeting of miRNA promoter regions by altered transcription factors or oncoproteins, and finally, dysregulated miRNAs processing [9].

### Abnormal miRNA expression and function in acute myeloid leukemia

The molecular and cytogenetic criteria currently defined by 2016 WHO is the most widely used diagnostic tool for AML [10]. Each AML subtype seems to exhibit a unique miRNA signature that distinguishes it from others. For example, Chen et al. reported miR-9, an oncogenic miRNA, was overexpressed in the mixed lineage leukemia (*MLL*)-rearranged AML patients. Inhibition of miR-9 expression could significantly reduce cell growth/viability and promote apoptosis [11]. Emmrich et al. found miR-9, significantly downregulated in pediatric AML with t(8;21), was characterized by its tumor-suppressive property. Upregulation of miR-9 decreased leukemic growth and induced monocytic differentiation of t(8;21) AML cell lines in vitro and in vivo. Functionally, miR-9 exerted its effects by binding to let-7 to suppress the oncogenic *LIN28B/HMGA2* axis [12]. In another study, miR-9-1 was observed to be downregulated in t(8;21) AML. Besides, overexpressed miR-9-1 induced differentiation and inhibited proliferation in t(8;21) AML cell lines [13]. MiR-10a/b was significantly increased in AML patients with t(8;21), t(9;11), *NPM1* mutation, and particularly M1, M2, and M3 subtype. Abnormal high expression in those patients led to unlimited proliferation of immature blood progenitors and repressed differentiation and maturation of mature blood cell [14]. Another study showed that miR-10a overexpression was significantly associated with French-American-British(FAB)-M3/t(15;17) subtypes and *NPM1* mutation, leading to the lower percentage of bone marrow (BM) blasts, while overexpression of miR-10b was correlated with *NPM1* and *DNMT3A* mutations, resulting in higher percentage of BM blasts [15]. Some studies observed overexpression of the miR-181 in cytogenetic normal AML (CN-AML) patients with *CEBPA* mutations, *FLT3-ITD*, and/or wild-type *NPM1* and t(15;17) [16–19]. MiR-155 was upregulated in *FLT3-ITD*-associated AML and targeted the myeloid transcription factor *PU.1*. Knockdown of miR-155 could repress proliferation and induce apoptosis of *FLT3-ITD*-associated leukemic cells [20].

MiRNA expression is also associated with morphologic sub-types of AML. MiR-122 expression, as an oncogene, was decreased in BM samples from pediatric patients with FAB subtype M7, and the forced expression of miR-122 in AML cell lines significantly inhibited cell proliferation and reduced the ratio of S-phase cells [21]. Xu et al. recently reported higher expression of miR-196b was observed in pediatric AML with M4/5 subtypes and predicted a poor outcome [22]. Another study compared M1 with M5 samples and noted that expressions of miR-146a/b, miR-181a/b/d, miR-130a, miR-663, and miR-135b were higher in M1, whereas expressions of miR-21, miR-193a, and miR-370 were higher in M5 [23]. Interestingly, in normal BM, miR-181a was enriched in B cells, T cells, monocytes, and granulocytes [24], but its overexpression was less common in monocytic lineage AML subtypes M4 or M5, but more so in M1 or M2 subtypes [25]. The expression levels of miR-195 in both BM and serum were significantly decreased, and pediatric patients with low serum miR-195 level more often had FAB-M7, unfavorable karyotypes, and shorter relapse-free and overall survival (OS) [26].

Changes in miRNA expression levels alter the expressions of downstream genes, promoting AML leukemogenesis [27]. For example, miR-155, acting as an oncogenic miRNA, may participate in the pathogenesis of AML by targeting *SHIP1* and downregulating transcription factor *PU.1* expression [28, 29]. This miRNA was regulated by *NF-κB*, whose activity was partly controlled by the *NEDD8*-dependent ubiquitin ligases [30, 31]. Schneider et al. reported that miR-155 expression was positively correlated with *Meis1* expression level in *MLL*-rearranged AML and first indicated that the transforming efficacy of *MLL*-fusions remained unaltered in the absence of miR-155, while knocking out miR-155 did not affect in vitro leukemia formation or progression [32]. Other studies demonstrated that miR-9/9* was aberrantly expressed in myeloid progenitors of most AML cases to inhibit neutrophil differentiation by regulating *EGN* post-transcriptional level. Moreover, miR-9 could promote proliferation of leukemia cells in adult CD34^+^ AML with normal karyotype by suppressing *Hes1* expression and knockdown of miR-9 could reduce circulating leukemic cell counts in peripheral blood (PB) and BM, attenuate splenomegaly and prolong survival in a xenotransplant mouse model [33, 34]. Li et al. showed that miR-193a expression was downregulated in *AML1/ETO*-positive leukemia cells because *AML1/ETO* triggered the heterochromatic silencing of miR-193a by binding at *AML1*-binding sites and recruiting chromatin-remodeling enzymes. Then the epigenetic silencing of tumor suppressor gene miR-193a led to leukemogenesis in AML with t(8;21) by activating the *PTEN/PI3K* signal pathway [35]. The latest study found that *Erbin* was the target of miR-183-5p that negatively regulated the *Erbin* expression, resulting in enhanced cell proliferation of AML cells via activation of *RAS/RAF/MEK/ERK* and *PI3K/AKT/FoxO3a* pathways [36]. MiR-125b, as an oncogenic miRNA, frequently overexpressed in human AML, could promote *MLL-AF9*-driven murine AML by *TET2-VEGFA* pathway. Zhang et al. reported that miR-203 downregulation frequently occurred in CD34 ^+^ AML cells in relation to CD34^−^ cells isolated from patients. Additionally, re-expression of miR-203 inhibited cell proliferation, self-renewal, and sphere formation in LSCs by targeting *survivin* and *Bmi-1* [37].

### MicroRNAs are associated with chemoresistance of AML

Chemoresistance is commonly seen in refractory and recurrent AML. Studies have shown that miRNAs are involved in AML chemotherapy resistance in many ways, such as apoptosis, cell cycle and ATP-binding cassette (ABC) transporter-mediated multidrug resistance.

Li et al. reported that miR-181a expression level was lower in the K562/A02 cells than in the K562 cells and could reduce doxorubicin resistance of K562/A02 cells by directly targeting the 3′-UTR of *BCL-2* and *MCL-1* mRNAs [38]. Similarly, miR-181a was underexpressed in the HL-60/Ara-C cell line compared with HL-60 cell line, while upregulated miR-181a in HL-60/Ara-C cells sensitized the cells to Ara-C treatment and promoted apoptosis by releasing cytochrome C and activating *caspase-9*/*caspase-3* pathway. Functionally, *BCL-2* was confirmed as a direct miR-181a target [39]. MiR-182-5p expression levels were higher in blood samples of AML patients than the normal samples. Cellular function indicated miR-182-5p inhibition in AML cells could decrease cell proliferation, promote AML cell apoptosis, and reverse cisplatin (DDP) resistance via targeting *BCL2L12* and *BCL2* expression [40].

Clinical chemotherapy drugs mainly interfere with cell cycle by inhibiting cellular DNA and RNA synthesis. *FoxM1*, an established oncogenic factor promoting cell cycle progression, plays a role in this process. MiR-370 expression was decreased in both leukemia cell lines (K562 and HL-60) and primary leukemic cells from patients BM with de novo AML. Ectopic expression of miR-370 in HL60 and K562 cells arrested cell growth and led senescence, while knockout of miR-370 expression promoted the proliferation of those leukemic cells. Mechanistically, miR-370 played a tumor suppressive role by targeting *FoxM1*. Moreover, when AML cells were treated with 5-aza-2′-deoxycytidine (a DNA methylation inhibitor), upregulation of miR-370 expression was observed, suggesting epigenetic silencing of miR-370 in leukemic cells [41]. *Cyclin D1* is a target protein of *PTEN* signaling pathway. *PTEN* mainly negatively regulates *PI3K/AKT* pathway through lipid phosphatase activity, then degrades *Cyclin D1*, leading to cell cycle organization in G1 phase. MiR-21 may desensitize leukemia cells to chemotherapy by interfering *PTEN* expression. Bai et al. reported high miR-21 expression in daunorubicin (DNR) resistant cell line K562/DNR. K562/DNR cell line stable transfected with miR-21 inhibitor was induced drug resistance, while inhibition of miR-21 enhanced cell sensitivity to cytotoxicity. Drug resistance mechanism of miR-21 was associated with regulating *PTEN* protein expression [42].

Chemotherapy drug resistance is also associated with efflux of hydrophobic drugs out of cells. ABC transporter and P-glycoprotein (*P-gp*), encoded by the *MDR1* gene, play pivotal roles in this process [43, 44]. MiR-381 and miR-495 were strongly underexpressed in K562/ADM cells. Restoring expression of miR-381 or miR-495 reduced expression of the *MDR1* gene and its protein product *P-gp*, and increased drug uptake via targeting the 3′-UTR of the *MDR1* gene [45]. In the drug-resistant cell line HL-60/VCR, miR-138 was significantly downregulated. Enhanced miR-138 expression significantly downregulated *P-gp* expression level and *MRP1* transcription to promote doxorubicin-induced apoptosis and reversed HL-60/VCR resistance to *P-gp* dependent and *P-gp* independent to drug delivery [46]. Besides, Feng et al. found that the expression of miR-331-5p and miR-27a was negatively correlated with *MDR1* expression, and the upregulation of miR-331-5p and miR-27a decreased *MDR1* expression and increased the sensitivity of K562-resistant cell line to doxorubicin [47].

### MicroRNAs and DNA methylation

Aberrant DNA methylation is an important epigenetic modification in the pathogenesis of AML. DNA methyltransferases are mainly divided into two types: *DNMT1* and *DNMT3*. The former maintains methylation, and the latter performs de novo methylation [48]. Garzon et al. demonstrated that miR-29b directly targeted *DNMT3*A and *DNMT3*B and indirectly targeted *DNMT1*, leading to DNA hypomethylation and tumor suppressor gene reactivation [49]. The indirect inhibition of *DNMT1* was mediated by a zinc finger-like structural transcription factor *SP1*, which bound directly to the *DNMT1* promoter region to start transcription [50]. MiR-29b downregulates *SP1* expression, thereby disrupting *SP1*-dependent *DNMT1* transcription [11]. Another example of *DNMTs* inhibition was hypomethylating tumor suppressor *P115INK4b* which could reduce susceptibility to myeloid leukemia in mouse model [51]. Phase 2 data of decitabine in elderly AML patients confirmed that miR-29b upregulation in BM cells could reduce the expression of *DNMTs*, enhance the effect of DNA hypomethylating agents, and therefore improve the remission rate [52].

MiR-29b could, however, be downregulated by *SP1*, as well as *KIT*. *KIT* overexpression has been observed in various tumors, including AML, and it promotes malignant cell proliferation [53]. Liu et al. identified that aberrant activation of *KIT* resulted in decreased *MYC*-dependent miR-29b expression and increased *SP1* expression, the latter then interacted with the *NF-κB/HDAC* complex to further inhibit miR-29b expression and transactivate *KIT* [54].

Contrary to miR-29b, which suppressed leukemogenesis, miR-221 was able to contribute to the aggressive nature of AML via the *NCL/miR-221/NF-κB/DNMT1* network. A group in China designed a nanoparticle that delivered anti-miR-221 antisense RNA in to leukemia cells. The nanoparticle could directly reactivate tumor suppressor gene *p27Kip1* by annihilating miR-221 and upregulate other tumor suppressor gene expressions by downregulating *DNMT1*. In mouse model, the nanoparticle showed promising therapeutic outcome [55].

Gene targets of miRNA may overcome the suppression or even downregulate the respective miRNA by DNA hypermethylation. For example, miR-375 could suppress *HOXB3* expression and cause AML cell proliferation arrest and colony reduction. In return, *HOXB3* enhanced *DNMT3*B’s binding to the promoter of miR-375, leading to DNA hypermethylation and lower expression of miR-375 [56].

### The role of exosomal microRNAs in acute myeloid leukemia

Exosomes are cell-derived, biologically active membrane-bound vesicles. The role of exosomes in hematopoiesis is receiving increasing attention. In 2015, Hornick et al. identified a set of miRNAs enriched in AML exosomes from the NOD/SCID/IL-2rγ^null^ (NSG) mice serum, such as let-7a, miR-99b, miR-146a, miR-150, miR-155, miR-191, and miR-1246. These serum exosomal miRNAs could potentially be used for early detection of AML [57]. Barrera-Ramirez et al. later sequenced miRNAs from exosomes isolated from AML patients’ marrow samples and from healthy controls. Of the five candidate miRNAs identified by differential packaging in exosomes, miR-26a-5p and miR-101-3p were significantly increased in AML, while miR-23b-5p, miR-339-3p, and miR-425-5p were significantly decreased, but the role and target genes of these exosomal miRNAs were still unknown [58]. Some of them might be AML tumor suppressors. Another study found that exosomes isolated from cultured AML cells or AML mice plasma were enriched with miR-150 and miR-155. Hematopoietic stem/progenitor cells (HSPCs) co-cultured with either of the two exosomes experienced impaired clonogenicity through the miR-150- or miR-155-mediated suppression of *c-MYB*, a transcription factor involved in HSPC differentiation and proliferation [59]. Moreover, Huan et al. found that the *Molm-14* exosome was also enriched in miR-150. This exosome was responsible for decreasing migration of AML cell lines and reducing the surface expression of *CXCR4* [60].

Some exosomal miRNAs may promote AML leukemogenesis. In a recent study, miR-7977 was found to have higher levels in AML exosomes than those from normal CD34^+^ cells. It might be a critical player in disrupting normal hematopoiesis via suppression of poly(rC)-binding protein. It also induced aberrant production of hematopoietic growth factors in mesenchymal stem cells, resulting in a hostile microenvironment for the normal stem cells [61].

Leukemia stem cells (LSCs) are believed to be the primary source of exosomes. Shedding harmful miRNAs via exosomes might be a mechanism of LSCs’ self-protection. Peng et al. discovered that miR-34c-5p was significantly downregulated in AML (excluding APL) stem cells compared to normal HSPCs. Increased expression of miR-34c-5p could induce LSC senescence ex vivo via both p53-dependent and independent *CKD/Cyclin* pathways. LSC could generate miR-34c-5p deficiency by actively packing and transporting miR-34c-5p out of the cells in exosomes. In return, miR-34c-5p could suppress exosome-mediated transfer via a positive feedback loop through *RAB27B*, a molecule that promotes exosome shedding. By targeting *RAB27B*, miR-34c-5p could enrich its intracellular level and induce LSC senescence [62].

### MicroRNAs as biomarkers for prognosis in acute myeloid leukemia

miRNAs have many properties of good AML prognostic biomarkers, such as wide presence in various tissues, highly conserved sequences, and easy and sensitive detection, as well as stability under extreme conditions [63, 64]. Mounting studies have shown that miRNAs can be used to predict outcome in CN-AML. Zhang et al. reported miR-216b overexpression as an independently poor prognostic factor for CN-AML and may provide a valuable biomarker associated with disease recurrence in AML [65]. In 224 patients with CN-AML, high miR-362-5p expression was associated with older age and shorter OS compared with low expressers [66]. Diaz-Beya et al. reported that high miR-3151 expression was commonly found in AML patients and obtained shorter disease-free, OS, lower CR rate and higher cumulative incidence of relapse compared with low expressers [67]. The underexpression of miR-328 in AML patients had poor clinical outcome and may provide a diagnostic and prognostic biomarker [68]. MiR-34a expression was negatively correlated with aggressive clinical variable. Patients with low miR-34a expression showed shorter overall and recurrence-free survival [69]. Xu et al. reported miR-135a as an independent prognostic factor for outcome in AML and a tumor suppressor in AML by inversely regulating *HOXA10* expression [70]. Moreover, patients with high expression levels of miR-146a and miR-3667 tended to have more favorable prognoses than their low expression counterparts [71], while underexpression of miR-122, miR-192, miR-193b-3p, miR-204, and miR-217, as well as miR-340 had been well studied to be unfavorable prognostic predictors of AML [72–77].

Some polymorphic miRNAs only had prognostic impact in certain subtypes. MiR-204 has two sites of variations: one is the upstream flanking region (rs718447 A > G), and the other is the gene itself (rs112062096 A > G). Butrym et al. demonstrated that miR-204 rs718447 GG homozygosity was a risk factor and associated with short survival [78].

Some miRNAs biomarkers might be helpful in selecting patients for allogenic hematopoietic stem cell transplant (allo-HSCT). High miR-425 level was associated with significantly longer OS and event-free survival (EFS) in non-transplant patients, but this association was not observed in post allo-HSCT patients. Instead, patients with downregulated miR-425 did better if they had allo-HSCT, suggesting that low miR-425 level might be an indication for transplant [79]. Overexpression of miR-99a predicted adverse prognosis in AML patients irrespective of transplant status, necessitating the investigation of novel alternative treatment in miR-99a overexpressors [80]. Moreover, high expression of miR-98 correlated with good clinical outcome in AML patients treated with chemotherapy alone [81].

miRNAs have potential prognostic value complementing information gained from gene mutations. MiR-181 family, which has been associated with *CEBPA* mutations and *FLT3-ITD* and/or *NPM1* wild-type in CN-AML, did demonstrate prognostic value [17]. Marcucci et al. reported favorable clinical outcomes in CN-AML patients with miR-181 overexpression and *CEBPA* mutations or miR-181 overexpression with *FLT3-ITD* [82]. In BM mononuclear cells of 113 de novo AML patients, miR-19b overexpression had more frequently occurred and high miR-19b expression had a higher frequency of mutations of *U2AF1* and *IDH1/2* genes and associated with poor prognosis and disease recurrence in AML [83]. AML patients with low miR-186 expression were frequently observed, and harbored lower complete remission rate and shorter OS, while miR-186^high^ patients had a significantly higher frequency of *CEBPA* mutation [84]. These findings suggested that measuring miRNA may have potential advantages for predicting prognosis of AML compared to assessed gene mutations such as *DMNT3A*, *FLT3-ITD*, *NPM1*, and *CEBPA*. In published studies, univariate and multivariate analysis showed that miR-98, miR-99a, miR-340, miR-216b, and miR-34c had independent stronger prognostic impact on EFS and OS (*P* < 0.05) than gene mutations in *FLT3-ITD*, *NPM1*, *DMNT3A*, *RUNX1*, *CEBPA*, and *TP53* [80, 81, 85, 86].

To summarize, miRNA researches in AML have yielded important results. The major miRNAs and their roles in AML were listed in Table 1.

**Table 1:** miRNAs in acute myeloid leukemia

miRNAs	Genetic abnormalities	Altered expression	Targets	Function	Reference
miR-9	t(8;21)(q22;q22.1) RUNX1-RUNX1T1; mutated NPM1; biallelic mutations of CEBPA	↑in MLL-rearranged AML	RHOH RYBP	miR-9 was upregulated by MLL-AF9 and increased MLL-AF9-mediated cell transformation in murine hematopoietic progenitor cells in vitro and in vivo. Mice transplanted with BM progenitors that overexpressed both MLL-AF9 and miR-9 (MLL-AF9+ miR-9) had higher frequency of c-Kit+ blast cells in the BM, spleen, and peripheral blood than MLL-AF9 mice. Moreover, MLL-AF9+ miR-9 leukemic cells had a higher frequency of immature blasts	[11]
		↓in t(8;21) AML	HMGA2 LIN28B	Increase proliferation and decrease monocytic differentiation	[12]
		↓in RUNX1-RUNX1T1(+)AML	RUNX1, RUNX1T1, RUNX1-RUNX1T1	RUNX1-RUNX1T1 triggered the heterochromic silencing of miR-9-1, resulting in hypermethylation of the miR-9-1 promoter in t(8; 21) AML. Silencing of miR-9-1 promoted expression of target genes(RUNX1, RUNX1T1, and RUNX1-RUNX1T1), which inhibited differentiation and promoted the proliferation of t(8; 21) AML cell lines	[13]
		↑3YPERLINK \lline	ERG	ERG is a direct target of miR-9 which contributed to miR-9/9*-induced differentiation of progenitor cells towards neutrophils	[33]
		↑3YPERLINK \l "_ENREF_33" \o "Nowek K, 2016 #298" hor><Yeaparients with normal karyotype	Hes1	miR-9 negatively regulated Hes1 expression and knockdown of miR-9 suppressed the proliferation of AML cells by the induction of G0 arrest and apoptosis in vitro, decreased circulating leukemic cell counts in peripheral blood and bone marrow, attenuated splenomegaly, and prolonged survival in a xenotransplant mouse model	[34]
		↓in AE-positive cell lines	SIRT1	Knockdown of SIRT1 expression inhibits cell proliferation in AE-positive AML cell lines	[87]
		↓in EVI1-induced AML	FOXO1 FOXO3	Increase proliferation and decrease monocytic differentiation	[88]
miR-21	Mutated NPM1; mutated RUNX1	↑in K562/DNR	*PTEN*	Decreased cell sensitivity to daunorubicin	[42]
		↑in SKM-1 cell	*PTEN*/AKT pathway	Downregulation of miR-21 expression inhibits proliferation and induces G1 arrest and apoptosis in SKM-1 cell	[89]
miR-22		↓iR-22LINK \l "	CRTC1 FLT3 MYCBP	Represses the CREB and MYC pathways	[90]
miR-29b	PML-RARA; mutated NPM1	↑in K562 cells	*DNMT3*A *DNMT3*B *DNMT1*	Increase DNA methylation and hypermethylation	[49]
		↓in t(8;21) AML	*SP1*	Upregulate KIT contributing to malignant proliferation	[54]
		↓in various subtypes of AML	AKT2 CCND2	Increase cell growth, leukemic progression in vivo	[91]
		↓in various subtypes of AML	MCL-1 CXXC6 CDK6	Increase cell growth, decrease apoptosis, leukemic progression in vivo	[92]
		↓in various subtypes of AML	*SP1* *DNMT3*A *DNMT3*B	Results in global DNA hypermethylation	[93]
		↑in NK cells		Damage to NK cells development and function	[94]
miR-34a	Biallelic mutations of CEBPA	↓in CEBPA mutated AML	E2F3	Increase proliferation and decrease differentiation	[95]
		↓in de novo AML	PDL1	Immune dysregulation	[96]
		↓in CEBPA mutated AML cell lines	HMGB1	Inhibit cell apoptosis and increased autophagy	[97]
miR-34b		↓iR-34bINK \l "_ENREF_	CREB	Survival signaling pathways	[98]
miR-34c-5p		↓in LSCs	RAB27B	Increase miR-34c-5p expression induced LSCs senescence ex vivo	
miR-99a	Mutated RUNX1; inv(16)(p13.1q22) or t(16;16) (p13.1;q22)			High miR-99a expression could predict worse outcome in AML patients undergoing allo-HCST	[80]
		↑in initial diagnosis and relapse		Regulate self-renewal, inhibiting differentiation and cell cycle entry	[99]
		↑in AML-AF9	SMARCA5 HS2ST3 HOXA1	Increase proliferation, colony formation, cell survival, inhibite differentiation	[100]
		↑in pediatric-onset AML (M1–M5)	CTDSPL TRIB2	Increase proliferation, colony formation, cell survival	[101]
miR-103		↑in K562 cells	COP1	Increase drug resistance of K562 cells to ADR	[102]
miR-125b	t(8;21)(q22;q22.1) RUNX1-RUNX1T1; PML-RARA; mutated NPM1	↑in MDS and AML with t(2;11) (p21;q23)		Inhibit differentiation	[103]
		↑in AML	LIN28A	Uncontrolled generation of myeloid cells	[104]
			IRF4	Induce myeloid leukemia in mice by inducing immortality, self-renewal, and tumorigenesis in myeloid progenitors	[105]
		↑in pediatric AML	FES PU.1	Block monocytic differentiation of AML in vitro	[106]
		↑in AML cell lines	NF-κB	Inhibits human AML cells invasion, proliferation and promotes cells apoptosis	[107]
miR-126	t(8;21)(q22;q22.1) RUNX1-RUNX1T1; PML-RARA; mutated NPM1	↑in t(8;21) and inv(16) AML	PLK2	Inhibits cell apoptosis and increase cell viability	[108]
		↑in LSCs of AML		Increase leukemic growth, and survival of leukemic stem and progenitor cells in vivo	[109]
		↑in t(8;21) AML	ERRFI1 SPRED1 FZD7	Both gain and loss of function of miR-126 promotes leukemogenesis in vivo through targeting distinct gene signaling	[110]
		↑in LSC of CN-AML		Increase LSC maintenance and self-renewal	[111]
		↑in LSCs of AML	ADAM9, ILK, GOLPH3, CDK3, TOM1	Increase LSC maintenance and self-renewal, quiescence, chemotherapy resistance in vivo	[112]
		↑in AML cell lines	TRAF7	Suppresses apoptosis by downregulating TRAF7, which blocks the c-FLIP pathway	[113]
miR-135a		↓in AML	HOXA10	Overexpression of miR-135a inhibits the proliferation and cell cycle and promotes cellular apoptosis	[70]
miR-139-5p		↓iR-139-5p \l "_E	EIF4G2	Repressing the translation initiation, specifically inducing the translation of cell cycle inhibitor p27 Kip1	[114]
miR-143		↑inCD34 + HSPCs	ERK5	Increase granulocyte surface marker Ly6G and a more mature morphology toward granulocytes induces apoptosis	[115]
miR-144-3p		↑iR-144-3pnn JU, 2018 #227" e	NRF2	Antiapoptotic	[116]
miR-146a	t(8;21)(q22;q22.1)RUNX1-RUNX1T1; mutated NPM1	↓in del(5q) MDS	TIRAP TRAF6	Inappropriate activation of innate immune signaling in HSPCs and megakaryocytic abnormalities	[117]
		Knockout in del(5q)MDS/AML		Increase cell survival and proliferation of propagating cells through the TRAF6/p62/NF-κB complex	[118]
			IRAK1	miR-146a knockout mice develop myeloid and lymphoid malignancies	[119]
				miR-146a deletion leads to myeloproliferation in mice	
		Knockout in del(5q) MDS/AML		Co-deletion of TIFAB and miR-146a may cooperate to induce TRAF6 signaling contributing to ineffective hematopoiesis	[120]
				miR-146a/Traf6 axis controls autoimmunity and myelopoiesis in mice	[121]
		↑in elderly AML patients	CXCR4 Smad4	Suppress the migration abilities of leukemia cells and promote cell cycle entry in leukemia cells	[122]
miR-149-5p		↑iR-149	FASLG	Targeting FASLG led to suppression on cell apoptosis	[123]
miR-150	PML-RARA	↓in various subtypes of AML	NANOG	Increase proliferation, colony, and sphere formation, increase tumor growth in vivo	[124]
		↓in various subtypes of AML	EIF4B, FOXO4, PRKCA, TET3	Increase cell growth and inhibits apoptosis in vitro and in vivo	[125]
		enriched in Molm-14 exosomes	CXCR4	Decrease migration of Ba/F3 cells and the surface expression of CXCR4	[60]
miR-150 miR-155		enriched in exosomes isolated from cultured AML cells	c-MYB	Hematopoiesis is suppressed by releasing exosomes that contain miR-150/miR155 targeting c-MYB	[59]
miR-181a		↑iR-181aNK \l "_ENREF_59"AML patients	KRAS, NRAS, and MAPK1	Targeting the RAS-MAPK-pathway	[126]
miR-182-5p	PML-RARA; Mutated NPM1; FLT3-ITD	↑in AML cell lines and patients blood sample	BCL2L12 BCL2	Promote cell proliferation, and reverse cisplatin (DDP) resistance	[40]
		↑in APL	CEBPα	Induce apoptosis	[127]
miR-192		↓in various subtype of AML	CCNT2	Increase proliferation and cell cycling, decrease differentiation	[128]
miR-193a		↓iR-*AML1/ETO*-positive leukemia cells	*PTEN*/PI3K signal pathway	*AML1/ETO* triggers the heterochromatic silencing of microRNA-193a (miR-193a) by binding at AML1-binding sites and recruiting chromatin-remodeling enzymes, which expands the oncogenic activity of AML-ETO, resulting in leukemogenesis	[35]
miR-193b	Biallelic mutations of CEBPA; mutated NPM1	↓mutati	CCND1,KIT, KRAS, or SOS2	Apoptosis and a G1/S-phase block	[74]
miR-196b	t(9;11)(p21.3;q23.3) MLLT3-KMT2A; mutated NPM1	↑in MLL associated AML		Increase proliferation and survival, and decrease differentiation and replating potential	[129]
		↑in MLL-associated AML	HOXA9 Meis1 FAS	Inhibit differentiation, promote cell proliferation, and induce leukemic progression in mice	[130]
miR-204	Mutated NPM1	↑in AML cells	BIRC6	Lead to AML cell apoptosis	[131]
		↑in NPMC+ AML	HOXA10 Meis1		[132]
miR-221	t(8;21)(q22;q22.1) RUNX1-RUNX1T1; CBFB-MYH1; mutated NPM1	↑in AML	NCL/miR-221/NF-κB/*DNMT1* network	Involve in DNA hypomethylation	[55]
miR-223	t(8;21)(q22;q22.1) RUNX1-RUNX1T1; CBFB-MYH1; PML_RARA; mutated NPM1; mutated RUNX1	↓in t(8;21) AML		Myeloid differentiation block	[133]
		↓in various subtypes of AML	E2F1	Lead to AML cell apoptosis	[134]
		↓in AML with adverse prognosis		Impair differentiation	[135]
		↓in various subtypes of AML	FBXW7	Increase cell proliferation and enhance apoptosis	[136]
miR-339-5p		↓in AML cells	SOX4	Inhibit cell proliferation of AML cells	[137]
miR-345-5p	Mutated NPM1	↓in AML cell lines	AKT1/2	Facilitate the proliferation of leukemia cells	[138]
miR-370		↓iR-370	NF1	Activation of the RAS signaling pathway	[139]
miR-375		↓in AML	miR-375-HOXB3-CDCA3/ *DNMT3*B pathway	Involve in DNA hypomethylation	[56]
miR-7977		↑in AML cell lines		miR-7977 in extracellular vesicles may be a critical factor that induces failure of normal hematopoiesis via poly(rC) binding protein 1 suppression	[61]
miR-26a-5p, miR-101-3p		↑in exosomes derived from MSCs in AML patients			[58]
miR-23b-5p, miR-339-3p, miR-425-5p		↓in exosomes derived from MSCs in AML patients			[58]
let-7a, miR-99b, miR-146a, miR-150, miR-155, miR-191, miR -1246		Enriched in exosomes from NSG mice serum			[57]
Let-7c		↓in AML patients with t(8;21) and inv(16)	PBX2	Promotes granulocytic differentiation	[140]

*Abbreviations*: *HSPC* hematopoietic stem and progenitor cell, *LSC* leukemia stem cells, *MSCs* bone marrow mesenchymal stromal cells, *NSG* NOD/SCID/IL-2rγnull, *allo-HSCT* allogeneic hematopoietic stem cell transplantation, *PB* peripheral blood, *BM* bone marrow

## Circular RNAs

Circular RNAs (circRNAs) are ubiquitous, stable, and conserved non-coding RNAs. They are closed circular RNA molecules and lack the 3′- and 5′-ends, different from the linear RNAs [141]. This structure was first described in viroids but later was also found in eukaryotic cells [142]. There are four types of circRNAs, namely exonic circRNAs (ecircRNAs), circRNAs from introns, exon-intron circRNAs (EIciRNAs), and intergenic circRNAs [143].

### Aberrant circRNA expression levels in acute myeloid leukemia

With the help of sequencing technology, more than 10,000 circRNAs in human have been identified [144, 145]. Aiming to pinpoint circRNAs that correlated with AML, Li et al. [146] used circRNAs microarray and characterized the expression profile of circRNAs in CN-AML, in which 147 circRNAs were upregulated and 317 circRNAs were downregulated compared with healthy control. An interesting phenomenon was that while hsa_circ_0004277 was one of the most significantly downregulated circRNAs in AML, its expression level was restored in patients who achieved complete remission, and the level post-remission was the same as healthy control, but it significantly dropped if the patient became relapse-refractory. Their findings suggested that hsa_circ_0004277 could be a potential diagnostic biomarker in detecting early relapse. Another circRNA, circPVT1, was overexpressed in AML harboring oncogene *MYC* amplification [147], and this association could hint that circPVT1 might impact the survival of AML patients.

In vitro and in vivo experiments have confirmed that the fusion circRNAs are derived from a fusion gene produced by chromosomal translocation. The study by Guarnerio et al. discovered *PML/RARα-*derivative f-circPR, and *MLL/AF9-*derivative f-circM9, and both promoted malignant transformation, chemoresistance, and leukemia cell survival [148]. *AML1* transcription factor complex is the most common target for leukemia-associated chromosomal translocations. *HIPK2* is part of the *AML1* complex and activates *AML1*-mediated transcription. Li et al. screened mutations of the *HIPK2* gene in 50 cases of AML and found two missense mutations (R868W and N958I) of *HIPK2* that are localized to nuclear regions with conical or ring shapes [149]. Hirsch et al. detected circular RNAs of *NPM1*. They found that the circular *NPM1* transcript, i.e., has_circ_0075001, had lower expression in healthy volunteers than in AML cell lines, and its expression was positively correlated with total *NPM1* expression, but not with the status of *NPM1* mutation [150]. Nevertheless, none of the current studies have elucidated the role of circRNA in AML pathogenesis.

The AML-related circRNAs and their roles in AML have been summarized in Table 2.

**Table 2:** CircRNAs in acute myeloid leukemia

circRNAs	Altered expression	Targets	Function	Reference
f-circPR	↑in NB4 cells		Promote proliferation and colony formation of leukemia cells	[148]
f-circM9	↑in THP-1 cells and K562 cells		Promote proliferation and colony formation of leukemia cells; knockout of f-circM9 increased apoptosis of THP1	[148]
hsa_circ_0075001	↑in AML(M0 or M1)↓in AML(M2, M4 and M5)		Hsa_circ_0075001 expression relates positively to total NPM1 expression, independent of the NPM1 mutational status; high hsa_circ_0075001 expression decreased expression of components of the Toll-like receptor signaling pathway	[150]
circ-ANAPC7	↑in AML patients BM	miR-181 family	Unknown	[151]
circ-100290	↑in BM cells from AML patients and AML cell lines	miR-203	Increase cell proliferation and inhibited apoptosis via interacting with miR-203/Rab10 axis	[152]
circPAN3	↑ircPAN3138" \o "Fan H, 2018 #193" or>Fan H</Author>< ↑ircPAN3138" \o "Fan H, 2018 #193" or>Fan H</Author><Year>2018	miR-153-5p miR-183-5p XIAP	Downregulation of circPAN3 by siRNA restores ADM sensitivity of THP-1/ADM cells depend on miR-153-5p/miR-183-5p-XIAP axis	[153]
circ_0009910	↑irc_000perients BM	miR-20a-5p	Promoted cell proliferation, inhibited apoptosis and predicted adverse prognosis	[154]
circ-HIPK2	Mutation of HIPK2 in AML and MDS		Impair AML1- and p53-mediated transcription	
	↓in APL patients PB and NB4 cells	miR-124-3p	Influence ATRA-induced differentiation of APL cells	[155]
circ-DLEU2	↓in pediatric AML-M5		Hypermethylation of DLEU2 affected prognosis	[156]
	↑in CN-AML patients BMand AML cell lines	miR-496	Promote AML cells proliferation and inhibited cell apoptosis and AML tumor formation in vivo via suppressing miR-496 and promoting PRKACB expression	[157]
has_cir_0004277	↓in mononuclear cells from AML patients BM		Increasing level of hsa_circ_0004277 is associated with chemotherapy	[158]
circPVT1	Overexpression in AML-amp		Unknown	[147]

*Abbreviations*: *amp* amplicons involving chromosome band 8q24, *BM* bone marrow, *PB* peripheral blood, *CN-AML* cytogenetically normal AML, *THP-1/ADM cell* doxorubicin (ADM)-resistant THP-1 AML cell

## Long noncoding RNA

Long noncoding RNAs (lncRNAs) are noncoding RNAs that are more than 200 nucleotides in length and lack a meaningful open reading frame [159]. lncRNAs are classified into intergenic lncRNAs, intron lncRNAs, sense lncRNAs, and antisense lncRNAs [160]. In cells, different lncRNAs may act as (1) a signal molecule, expressed at specific time and in specific tissues, regulating the expression of certain genes; (2) a miRNA sponge; (3) a leader molecule, directing RNAs that bind to RNA-binding proteins to reach regulatory sites, and regulating the expression of the relevant gene; and (4) a scaffold molecule, being a central platform for the assembly of other molecules.

### lncRNAs involved in acute myeloid leukemia pathogenesis

lncRNAs play an important role in BM cell differentiation and are subjected to differentiation-inducing therapies. HOTAIRM1 and NEAT1 are two important examples. HOTAIRM1 is a myeloid-specific lncRNA that is transcribed from the locus between the *HOXA1* and *HOXA2* genes. In the initial studies of lncRNAs in AML, HOTAIRM1 was found to be a regulator of myeloid differentiation and maturation by affecting the expression levels of integrin genes such as *ITGA4*(*CD49d*) and *ITGAX*(*CD11c*)*.* Knocking down HOTAIRM1 would prohibit all-trans retinoic acid (ATRA)-induced granulocyte differentiation [161]. The fact that HOTAIRM1 came from the *HOXA* cluster might imply that it could regulate nearby genes in the HOXA cluster, although this warranted further investigation. The other lncRNA, NEAT1, was significantly downregulated by *PML-RARα* in de novo APL samples compared with those of healthy donors. In NB4 cells, silencing NEAT1 could block ATRA-induced differentiation [162]. The roles of HOTAIRM1 and NEAT1 in normal hematopoiesis and leukemogenesis are awaiting further elucidation.

Other lncRNAs participate in regulating AML cell proliferation, cell cycle, and apoptosis. A typical example is lncRNA PVT1 [163]. The coding sequence of PVT1 on the chromosome is adjacent to *MYC*. Functional acquisition of *MYC* and *PVT1* due to amplification of 8q24.21 is observed in approximately 10% of AML patients [164]. In AML cell lines, overexpression of PVT1 could induce apoptosis and necrosis, probably through downregulating *c-MYC* expression [165, 166]. UCA1 is another lncRNA that might have the capability to modulate AML cell proliferation; silencing of UCA1 by short hairpin RNA would result in a significantly slower cell proliferation and G1 cell cycle arrest. UCA1 could promote proliferation by inhibiting the expression of the cell cycle regulator *p27kip1* [167]. Similarly, CRNDE could coordinate the proliferation and differentiation of AML cells as demonstrated by Wang et al. in their experiment with the U937 cell line [168]. At present, most of the lncRNA studies in AML are ex vivo, and the detailed mechanisms of lncRNA regulating cell proliferation remain to be investigated.

### LncRNA expression in AML with recurrent genetic mutations

Distinct lncRNA expression patterns have been observed in different AML subtypes, reflecting the heterogeneity of this disease. AML is most common in older patients (age ≥ 60) although they often have a worse prognosis [169, 170]. Numerous studies have identified characteristic lncRNA profiles in age ≥ 60 CN-AML patients with recurrent genetic mutations such as *FLT3-ITD*, *NPM1*, *CEBPA*, and *RUNX1* mutations.

#### *FLT3-ITD*-related lncRNAs

Wilms’ tumor 1(*WT1*) expression positively correlates with *FLT3-ITD* in patients with AML [171]. Benetatos et al. identified that lncRNA MEG3 could be activated by *WT1* and *TET2* and it acted as a cofactor of *WT1*, enhancing leukemogenesis [172].

#### *CEBPA* mutation-related lncRNAs

CCAAT/enhancer-binding protein-α (*CEBPA*) is a critical regulator of myeloid differentiation and 10% of AML have mutations in *CEBPA*, which may lead to the expression of a 30-kDa dominant negative isoform (*C/EBPα-p30*) [173]. Hughes et al. identified a *C/EBPα-p30* target lncRNA UCA1. It was increased in CN-AML patients with biallelic *CEBPA* mutations and could promote cell proliferation [167]. Another study reported that *HOXB-AS3* was the most downregulated lncRNA in *CEBPA*-mutated AML while it was upregulated in *NPM1*-mutated AML [174].

#### *NPM1* mutation-related lncRNAs

Besides the aforementioned *HOXB-AS3* [175], the coiled-coil domain containing 26 (CCD26) is also upregulated in the *NPM1*-mutated AML and is a retinoic acid-dependent modulator of myeloid cell differentiation and death [176]. Apart from them, a recent study employing RNA-sequencing identified another *NPM1* mutation-associated lncRNA XLOC_109948 whose high expression predicted a poor prognosis [177].

#### *RUNX1* mutation-related lncRNAs

Fernando et al. first characterized CASC15, a conserved lncRNA upregulated in pediatric AML with *RUNX1* mutation. High expression of CASC15 led to myeloid-predominant BM development, decreased engraftment, and colony formation. Researchers also found that CASC15 positively regulated *YY1*-mediated *SOX4* promoter [178].

### Prognostic value of lncRNAs in acute myeloid leukemia

LncRNA expression level could predict AML clinical features and outcomes. A published study has confirmed that lncRNAs can assist to predict clinical outcome in older patients with CN-AML. In the basic of 148 CN-older (age > 60 years) AML patients, Garzon et al. evaluated the associations of lncRNA expression with clinical characteristics, gene mutations, and outcome and built a lncRNA score including 48 lncRNAs for independently outcome prognosis [179]. Li et al. reported that SNHG5 overexpression was frequently observed in AML patients with advanced FAB classification and unfavorable cytogenetics. Furthermore, a higher SNHG5 expression level was also associated with shorter OS [180]. Yang et al. have determined the PANDAR expression level and its clinical significance in 119 de novo AML patients. AML patients expressing a higher level of PANDAR were associated with low complete remission rate and adverse prognosis in comparison with those with lower expression of PANDAR [181]. Moreover, high HOTAIR expression was associated with adverse clinical outcomes [182]. Based on 64 de novo non-M3 AML patients, Pashaiefar et al. found that low expression of IRAIN was independently associated with adverse prognosis: higher white blood cell count and blast counts and shorter OS and relapse-free survival. Besides, patients with refractory response to chemotherapies and those with subsequent relapse were more likely to show a lower initial IRAIN expression [183].

TUG1 has been in the spotlight of AML research. Higher TUG1 expression level occurred in AML patients with monosomal karyotype, *FLT3-ITD* mutation, and poor-risk and correlated with higher white blood cell counts and worse event-free survival and overall survival [184]. Luo et al. investigated the correlation of TUG1 expression with clinicopathological features and its predictive value for treatment response and survival profiles in refractory or relapsed AML patients age ≥ 60 years. They demonstrated that AML patients with higher TUG1 expression had shorter OS, and a lower rate of complete response and overall response than those with lower TUG1 expression [185].

Overall, there are only a few published reports of lncRNAs’ prognostic value in AML; thus, more profound works are required to investigate the association of lncRNAs, clinical characteristics, mutations, and outcome. The researches on AML-related lncRNAs are summarized in Table 3.

**Table 3:** lncRNAs in acute myeloid leukemia

lncRNAs	Altered expression	Targets	Function	Reference
PVT1	↑in AEL/APL		Protect MYC from degradation to promoted promyelocytes proliferation	[163]
CRNDE	↑in AML cell lines		Promote cell proliferation and arrest cell cycle in G0-G1 phase	[168]
MEG3	↓in AML		Promote AML leukemogenesis	[172]
CCD26	↑in NPM1-mutated AML	c-Kit	Control the growth of AML cells	[176]
H19	↑in AML-M2 patients	has-miR-19a/b	Regulated the expression of ID2 through competitive binding to miR-19a/b to increase cells proliferation	[186]
NEAT1	↓in AML blood sample and AML cell lines	miR-23a-3p	Increase myeloid cell proliferation and ATRA-induced myeloid differentiation, and induce apoptosis	[187]
UCA1	↑in AML cell lines and CN-AMLwith biallelic CEBPA	miR-126, RAC1	Increased cell proliferation, inhibited apoptosis, migration, and invasion by sponging miR-126	[188]
	↑in AML cell lines and CN-AML with biallelic CEBPA	p27kip1	Role in promoting cells proliferation is to sequester hnRNP I to inhibit the expression of the cell cycle regulator p27kip1	[167]
	↑in HL-60 and HL-60/ADR	miR-125a	Poor chemotherapy overcome	[189]
HOTAIR	↑in de novo AML patients	miR-193a;c-Kit	Increase AML cells proliferation, inhibited apoptosis and infiltration of leukemic blasts and number of AML cells colony formation, and shorten overall survival time	[190]
	↑in LSC	p15	Promote the self-renewal of leukemia stem cells	[191]
CCAT1	↑in HL60 and AML PB	miR-155, c-Myc	Upregulated c-Myc expression to increased cells proliferation and differentiation by its competing endogenous RNA (ceRNA) activity on miR-155	[192]
FTX	↑in U937 and THP-1	miR-342, ALG3	Drug resistance	[193]
PANDAR	↑ANDARLINK		Predict adverse prognosis in AML	[181]
HOXA-AS2	↑in APL	TRAIL-mediated pathway	Lead to fine-tuning of apoptosis during ATRA-induced myeloid differentiation	[194]
	↑00PERLINK \l "_ENREF_200" \o "Zhao H, 2013 #197" or><adriamycin-based chemotherapy and in U/A and T/A cells	miR-520c-3p/S100A4 Axis	Knockdown of lncRNA HOXA-AS2 inhibited ADR cell proliferation and chemoresistance of AML by the miR-520c-3p/S100A4 Axis, and promoted apoptosis	[195]
HOTAIRM1	↑in AML cell lines	HOXA1, HOXA4, CD11b,CD18,miR-20a/106b miR-125b	Regulate myeloid cell differentiation and cell cycle via enhancing the autophagy pathway and PML-RARα degradation	[161] [196] [197] [198]
IRAN	↑in AML	IGF1R	long-range DNA interactions	[199]
RUNXOR	↑in AML	RUNX1	Participate in chromosomal translocation	[200]
ANRIL	↑in AML patients at diagnosis ↓in patients after CR	ANRIL/AdipoR1/AMPK/SIR pathway	Promote cell survival	[201]
vtRNA2-1			Regulate pPKR	[202]
linc-223	↓in AML cell lines	IRF4; miR-125-5p	Control proliferation and differentiation of AML cells and IRF4 downregulation by binding miR-125-5p	[203]
LINC00899	↑INC00899K \l "_ENREF_13patients		As a novel serum biomarker for diagnosis and prognosis of AML	[204]

*Abbreviations*: *CR* complete remission, *PB* peripheral blood, *CN-AML* cytogenetically normal AML, (*U/A*) U937/ADR cell, (*T/A*) THP-1/ADR cell

## lncRNAs and circRNAs can interfere with miRNA function in AML

It has recently been learned that aberrant expression of lncRNAs and circRNAs in AML can change the function of specific miRNAs contributing to initiation, maintenance, and development of leukemogenesis.

In 2011, Salmena et al. proposed a competing endogenous (ceRNA) hypothesis that lncRNAs competitively binds to endogenous miRNAs in AML. A lncRNA, H19, for example, was found overexpressed in BM samples from patients with AML-M2; it promoted AML cell proliferation by sequestering miR-19a/b [186]. The lncRNA NEAT1 that competitively binds miR-23a-3p, an oncogenic miRNA, thus modulating the expression of *SMC1A* in AML cells, which affected myeloid leukemia cell proliferation and apoptosis [187]. UCA1 is a functional lncRNA that promoted cell proliferation, migration, and invasion of human AML cells via binding miR-126 [188]. In accord with Zhang et al.’s study, its expression was abnormally upregulated following doxorubicin-based chemotherapy and knockdown of UCA1 helped overcome chemoresistance in pediatric AML by suppressing glycolysis via binding miR-125a [189]. FTX is another lncRNA involved in chemoresistance, and it controlled the expression of *ALG3* by binding miR-342 [193]. HOXA cluster antisense RNA 2 (HOXA-AS2) was significantly upregulated in BM samples from AML patients after treatment with adriamycin-based chemotherapy and sponged miR-520c-3p to contribute to chemoresistance in AML [195]. An oncogenic activity of lncRNA was also shown by HOTAIR that regulating the expression of *c-Kit* in AML cells through competitively binding miR-193a, an important tumor-suppressor miRNA to predict a poor clinical outcome [190]. HOTAIRM1, a lncRNA located in the HOXA genomic region, is related to myeloid differentiation which sequestered miR-20a, miR-106b and miR-125b, all of which targets autophagy-associated genes, leading to the degradation of oncoprotein *PML-RARA*. Moreover, Chen et al. showed that CCAT1 is an oncogenic lncRNA that upregulated *c-Myc* via its ceRNA activity on miR-155 to repress monocytic differentiation and promote cell growth [192]. The host non-coding transcript of miR-223 of linc-223, found downregulated in AML, is a functional lncRNA which regulated proliferation and differentiation of AML cells by binding miR-125-5p [203].

In recent years, the research of circRNAs, as one of ncRNAs, is focused on their function as “miRNA sponges” in the complex endogenous RNA networks. A circRNA HIPK2, for example, sponged miR-124-3p to regulate the differentiation of all-trans retinoic acid (ATRA)-induced NB4 cells [155]. Chen et al. [151] reported that circANAPC7 was significantly upregulated in AML and used an Arraystar human circRNAs microarray and bioinformatics analysis to predict when ANAPC7 might bind miR-181 family to participate in AML pathogenesis. An oncogenic activity of circRNA was also shown by DLEU2, which was highly expressed in AML, that inhibited miR-496 expression to promote cell proliferation and inhibit cell apoptosis [157]. A circular RNA 100290, which as an oncogenic circRNA was upregulated in AML, showed that it sponged miR-203 to control AML cell proliferation and apoptosis [152]. Moreover, Shang et al. demonstrated the circRNA PAN3 controlled AML chemoresistance by sequestering miR-153-5p and miR-183-5p, [153]. Moreover, Ping et al. showed that circ_0009910, upregulated in AML BM and predicting adverse outcome of AML patients, sponged miR-20a-5p to promote cell proliferation and inhibit [154].

**Fig. 1:**
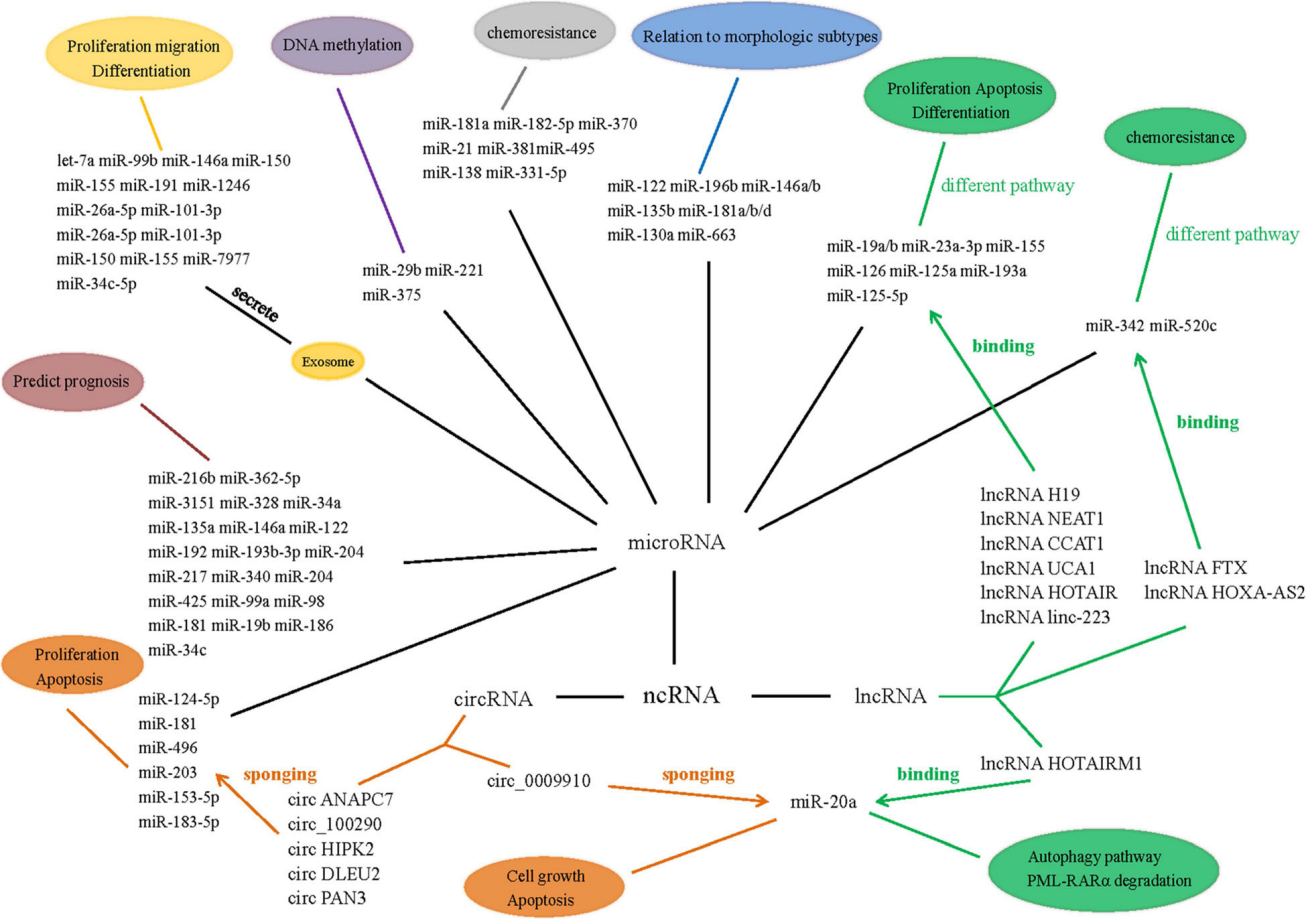
The connections of three ncRNAs involved in AML

In combination, lncRNAs and circRNAs introduce a complex layer in the miRNA target network, respectively, while lncRNA HOTAIRM1 and circ_0009910 can bind with the same miRNA, miR-20a, to play a different function in AML. The connections of these three ncRNAs involved in AML is shown in Fig. 1. But how lncRNAs and circRNAs compete with each other to bind with the same miRNAs remains unclear, thus making it necessary to further explore the relationship between lncRNAs and circRNAs in AML, to illustrate AML pathogenesis and therapy.

## Conclusion

ncRNAs are widely recognized as critical participators in AML pathogenesis. Indeed, specific ncRNA expression could assist clinicians to classify subtypes, to evaluate prognosis, and to predict the response of drug treatment in AML. In this review, we discussed miRNAs, circRNAs, and lncRNAs, involving in subtypes, molecular function, chemoresistance and prognosis in AML, and the interactions between three major ncRNAs. Currently, the role of miRNAs in AML is most studied, but the mechanisms of miRNAs in AML still remain complex and unclear owing to miRNAs target genes ranging from tens to hundreds and involving different signaling pathways. In recent years, lncRNAs and circRNAs are introduced into miRNA network one after another and can be used as ceRNA of miRNAs and miRNAs sponge to regulate miRNA expression in AML. In our review, we reported that some lncRNAs such as UCA1 and linc223 could target the same miRNA, miR-125, to control proliferation, apoptosis, and differentiation, and lncRNA HOTAIRM1 participated in autophagy pathway by binding with miR-125. MiR-125 has been reported to promote *MLL-AF9*-driven murine AML by *TET2-VEGFA* pathway and target autophagy-associated genes, leading to the degradation of oncoprotein *PML-RARA*. CircRNA_0009910 could also bind miR-20 via competing with lncRNA HOTAIRM1 to regulate proliferation and apoptosis. However, whether these three lncRNAs directly affect *MLL-AF9*-driven AML and autophagy, the target genes of miR-20 are not clear. Thus it is important to find the crossover miRNAs of the three ncRNAs to help illustrate the connections among these three ncRNAs. However, currently, there is very little literature on this subject and the connection networks of the three ncRNAs are required for further study. Subsequently, we will also trace relative studies and update the interaction networks of miRNAs, lncRNAs, and circRNAs.

## Abbreviations

3′-UTR: 3′-untranslated region
ABC: ATP-binding cassette
allo-HSCT: Allogenic hematopoietic stem cell transplant
AML: Acute myeloid leukemia
ATRA: All-trans retinoic acid
BM: Bone marrow
C/EBPα-p30: 30-kDa dominant negative isoform
CCD26: Coiled-coil domain containing 26
CEBPA: CAAT/enhancer-binding protein-α
ceRNA: Competing endogenous RNA
circRNA: Circular RNA
CN-AML: Cytogenetic normal AML
DNR: Daunorubicin
EFS: Event-free survival
EZH2: Zeste homolog 2
FAB: French-American-British
HOXA-AS2: HOXA cluster antisense RNA 2.
HSPCs: Hematopoietic stem/progenitor cells
LncRNA: Long noncoding RNA
LSCs: Leukemia stem cells
miRNA: MicroRNA
MLL: Mixed lineage leukemia
ncRNA: Noncoding RNA
OS: Overall survival
PB: Peripheral blood
P-gp: P-glycoprotein
WT1: Wilms' tumor 1
